# Evaluation of humoral immune response after yellow fever infection: an observational study on patients from the 2017–2018 sylvatic outbreak in Brazil

**DOI:** 10.1128/spectrum.03703-23

**Published:** 2024-03-21

**Authors:** Andreza Parreiras Gonçalves, Letícia Trindade Almeida, Izabela Maurício de Rezende, Jordana Rodrigues Barbosa Fradico, Leonardo Soares Pereira, Dario Brock Ramalho, Marcelo Antônio Pascoal Xavier, Carlos Eduardo Calzavara Silva, Thomas P. Monath, Angelle Desiree LaBeaud, Betania Paiva Drumond, Ana Carolina Campi-Azevedo, Olindo Assis Martins-Filho, Andréa Teixeira-Carvalho, Pedro Augusto Alves, Grupo de Estudos de Pesquisa e Resposta em Febre Amarela do Estado de Minas Gerais

**Affiliations:** 1 Instituto René Rachou, Fundação Oswaldo Cruz (FIOCRUZ-Minas), Belo Horizonte, Minas Gerais, Brazil; 2 Department of Pediatrics, Infectious Disease Division, Stanford University School of Medicine, Stanford, California, USA; 3 Departamento de Microbiologia, Instituto de Ciências Biológicas, Universidade Federal de Minas Gerais, Belo Horizonte, Minas Gerais, Brazil; 4 Hospital Eduardo de Menezes (HEM), Fundação Hospitalar do Estado de Minas Gerais (FHEMIG), Belo Horizonte, Minas Gerais, Brazil; 5 Departamento de Anatomia Patológica e Medicina Legal, Faculdade de Medicina, Universidade Federal de Minas Gerais, Belo Horizonte, Minas Gerais, Brazil; 6 Crozet BioPharma LLC, Lexington, Massachusetts, USA; Centro de Investigacion y de Estudios Avanzados del Instituto Politecnico Nacional, Mexico City, Mexico

**Keywords:** yellow fever, PRNT, neutralizing antibodies, wild-type strain, 17DD YFV strain, late relapsing hepatitis after yellow fever, YF immune response, sylvatic YF

## Abstract

Between 2016 and 2018, Brazil experienced major sylvatic yellow fever (YF) outbreaks that caused hundreds of casualties, with Minas Gerais (MG) being the most affected state. These outbreaks provided a unique opportunity to assess the immune response triggered by the wild-type (WT) yellow fever virus (YFV) in humans. The plaque reduction neutralization test (PRNT) is currently the standard method to assess the humoral immune response to YFV by measuring neutralizing antibodies (nAbs). The present study aimed to evaluate the humoral immune response of patients from the 2017–2018 sylvatic YF outbreak in MG with different disease outcomes by using PRNTs with a WT YFV strain, isolated from the 2017–2018 outbreak, and a vaccine YFV strain. Samples from naturally infected YF patients were tested, in comparison with healthy vaccinees. Results showed that both groups presented different levels of nAb against the WT and vaccine strains, and the levels of neutralization against the strains varied homotypically and heterotypically. Results based on the geometric mean titers (GMTs) suggest that the humoral immune response after a natural infection of YFV can reach higher levels than that induced by vaccination (GMT of patients against WT YFV compared to GMT of vaccinees, *P* < 0.0001). These findings suggest that the humoral immune responses triggered by the vaccine and WT strains of YFV are different, possibly due to genetic and antigenic differences between these viruses. Therefore, current means of assessing the immune response in naturally infected YF individuals and immunological surveillance methods in areas with intense viral circulation may need to be updated.

**IMPORTANCE:**

Yellow fever is a deadly febrile disease caused by the YFV. Despite the existence of effective vaccines, this disease still represents a public health concern worldwide. Much is known about the immune response against the vaccine strains of the YFV, but recent studies have shown that it differs from that induced by WT strains. The extent of this difference and the mechanisms behind it are still unclear. Thus, studies aimed to better understand the immune response against this virus are relevant and necessary. The present study evaluated levels of neutralizing antibodies of yellow fever patients from recent outbreaks in Brazil, in comparison with healthy vaccinees, using plaque reduction neutralization tests with WT and vaccine YFV strains. Results showed that the humoral immune response in naturally infected patients was higher than that induced by vaccination, thus providing new insights into the immune response triggered against these viruses.

## INTRODUCTION

Yellow fever (YF) is an infectious disease caused by the YF virus, *Orthoflavivirus flavivirus* (Flaviviridae family, *Flavivirus* genus). This enveloped and single-stranded RNA virus has an 11-kb genome, which encodes three structural (membrane, envelope, and capsid) and seven non-structural proteins. Yellow fever virus (YFV) is maintained in nature in a transmission cycle involving non-human primates and mosquitoes. YF major epidemics have been reported throughout history, and despite the availability of safe and efficient live, attenuated vaccines (manufactured using 17D-204 and 17DD strains, both derived from the 17D strain) since the 1930s, regular outbreaks still occur in Africa and South America, showing that YF is still a significant public health concern worldwide (1–3). The World Health Organization (WHO) estimates a global occurrence of 100,000 cases and approximately 30,000 deaths from YF annually, but because this disease is underreported, its actual incidence is still unknown (4). Recently, sylvatic YF outbreaks were recorded in previously non-endemic areas of South America, especially in the south and southeast regions of Brazil. A monophyletic lineage, identified as BR-YFV_2016/2018, within the genotype South America I, was identified as responsible for causing the outbreaks. This lineage was likely introduced from the midwest region of Brazil into the southeast region around 2015 and circulated persistently in the area up to 2018 (5). Between July 2016 and June 2018, 2,523 cases in non-human primates, 2,155 human cases, and 745 human deaths by YF were recorded in Brazil. The human cases happened mainly due to low vaccination coverage and had a major impact, being the most extensive outbreak within the last eight decades in Brazil (6–8).

The infection caused by the YFV is acute and can range from asymptomatic cases to severe, fulminant, and fatal presentations (1). Moderate and severe forms of YF are characterized by phases with non-specific symptoms. The first phase, called infection, happens during the first 3–6 days of symptom onset and is marked by the onset of fever, headache, asthenia, and myalgia, followed by a remission phase. Shortly after, about 15%–25% of patients exhibit a return and worsening of symptoms during intoxication, when severe fever, jaundice, and hemorrhagic manifestations are common and can last up to 8 days. During this phase, anti-YFV antibodies are measurable in serum. Up to 50% of patients who evolve to the intoxication phase will die. Surviving patients evolve to the convalescent phase, characterized by long periods of asthenia and myalgia lasting for months (1, 9). Recently, a phenomenon called late-relapsing hepatitis after yellow fever (LHep-YF) was described, affecting a small portion (approximately 16%) of patients in the last outbreak in Brazil. LHep-YF is characterized by a reoccurrence of jaundice and elevation in liver transaminases during the convalescent phase (from 46 to 90 days after symptom onset) after a previous complete resolution of symptoms (10–12).

A rapid, specific, robust, and durable immune response is triggered soon after the onset of YFV infection, mediated mainly by neutralizing antibodies (nAb) directed against the envelope protein (13–16). It has been demonstrated through studies using the 17D and 17DD vaccine strains that the YFV can induce the production of nAb in up to 90% of vaccinees 10 days after vaccination and in 95% after 30 days (2, 15, 17, 18). Infection with wild-type (WT) YFV and vaccine strains can induce the production of nAb; however, multiple passages in mice and eggs attenuating the 17DD strain led to multiple mutations. These mutations may affect the recognition of viral proteins by nAb, resulting in different humoral responses compared to responses against WT strains (13, 14, 19, 20). The same polymorphisms and differences in recognition by nAb have been observed in African YFV genotypes (West African I and II) that are more closely related to the vaccine strains compared to the South American YFV genotypes (I and II) (21). Compared to WT strains, changes in the envelope protein of the 17DD strain also resulted in changes in the entry pathways into cells and, consequently, in differences in the replicative profiles of these viruses (22). These may entail differences in the immune response triggered by these viruses, making it relevant to investigate viral and host characteristics to help elucidate the mechanisms that different YFV strains use to activate and modulate the immune response. The evaluation of humoral responses after natural YFV infection or immunization with YFV-17DD is crucial to better understand the immune response triggered by these viruses. For the humoral response analysis, the plaque reduction neutralization test (PRNT) is currently the gold standard for detecting and measuring anti-YF nAb and for assessing the protective humoral immunity following YF natural infection and vaccination (2, 23–25).

The impact of recent sylvatic YFV outbreaks and the high lethality rates and severity of YF reinforce its epidemiological relevance. Moreover, the current scarcity of studies on immunopathological aspects of this disease, together with peculiarities observed in recent outbreaks, such as the recently described cases of LHep-YF, has demonstrated the knowledge gap in the clinical and immunological characteristics of patients affected by this disease. Thus, this study aimed to evaluate the humoral immune response of patients from the last sylvatic YF outbreak in Brazil, with different clinical outcomes. A total of 59 patients who were diagnosed with YF during the 2017–2018 outbreak in Minas Gerais (MG), Brazil, with different clinical outcomes, were included in this study. Samples from 23 healthy individuals primovaccinated with a standard dose of the 17DD vaccine from a YF vaccine study performed in São Paulo, Brazil, were also included as controls. PRNT assays were performed to assess the prevalence and levels of nAb against both WT and vaccine strains of YFV in naturally infected YF patients compared to healthy vaccinated individuals.

## POPULATION, MATERIALS, AND METHODS

### Study population

Samples were obtained from patients who were diagnosed with YF during the 2017–2018 outbreak in MG, Brazil, and admitted to the Hospital Eduardo de Menezes in Belo Horizonte, MG, Brazil. All patients had the diagnosis confirmed through serological and/or molecular methods, and differential diagnoses confirmed through serological tests for dengue, Zika, chikungunya, hantavirus, hepatitis A, hepatitis B, and hepatitis C, Rocky Mountain spotted fever, and leptospirosis, performed by reference laboratories linked to the State Health Secretary of MG, Brazil (12, 26). Clinical (vaccination status, time of sample collection, and disease outcome) and demographic data (age and gender) were obtained from the patient’s medical records and used for descriptive analyses of the groups and to categorize the patients according to clinical outcome (hospital discharge, death, and occurrence or not of LHep-YF). All data collected from the patients is maintained on the Research Electronic Data Capture platform.

Seventy-two serum samples from 59 YF patients collected during the acute (1–15 days after symptom onset) and convalescent (15–120 days after symptom onset) phases of YF infection were selected and categorized into two groups according to disease outcome as follows: (i) 37 patients who survived and were discharged from the hospital [serum samples collected between 30 and 60 days post-infection (DPI)] and (ii) 22 patients who died during hospitalization due to YF complications (serum samples collected between 4 and 15 DPI). Samples from patients who were discharged were further categorized into three groups, according to the development or not of LHep-YF and to the time of sample collection, as follows: (i) 15 patients who survived the disease and developed LHep-YF (serum samples collected between 30 and 60 DPI); (ii) a subgroup of 13 patients, among the 15 who survived the disease and developed LHep-YF, who had paired serum samples available from different times of the convalescent phases of infection (serum samples collected between 85 and 120 DPI); and (iii) 22 patients who survived the disease and did not develop LHep-YF (NLHep-YF) (serum samples collected between 30 and 60 DPI). Both male (*n* = 51) and female (*n* = 08) patients were included, with ages ranging from 18 to 79 years. Of the 59 YF patients included in the study, 4 had been vaccinated with 17DD between 1 and 4 days before or after symptom onset. Confirmed vaccination records were unavailable for all YF patients evaluated in this study.

Additionally, serum samples from healthy YF vaccinees (*n* = 23) from a YF vaccine study performed in São Paulo, Brazil, were used as a control group for this study. These samples are from individuals primovaccinated with a standard dose of the 17DD vaccine and were collected with a comparable time interval between vaccination and blood sampling as the YF patients (from 30 to 60 days after immunization). The vaccinees were from a region without YFV circulation and had been previously screened for YF antibodies, and only those who were seronegative before vaccination were included in this study. Both male and female patients were included, although information about gender was not available for all participants from the control group, and their ages ranged from 18 to 59 years.

### Virus

The 17DD vaccine virus stock used for the PRNT was obtained after three passages in *Aedes albopictus* (C6/36) cells of the commercial vaccine sample produced by Biomanguinhos (Fundação Oswaldo Cruz, Rio de Janeiro, Brazil). Briefly, the vaccine was reconstituted and homogenized by vortex, and the liquid was transferred to a 1.5mL microtube. The vaccine virus was then titrated by plaque assay (27) and used in further experiments. The YFV WT sample (Hu-BR2018) was isolated from a serum sample collected from a YF patient from the 2017–2018 sylvatic outbreak in MG, during the acute phase of the disease, and passed 10 times in C6/36 cells. Additionally, limiting dilution and plaque purification assays (28, 29) were performed to ensure that the wild-type viral stocks produced homogeneous lysis plaques. Viral stocks were produced in C6/36 cells and titrated in Vero cells by plaque assay (27), and aliquots of both viruses were stored at −80°C. Both 17DD and the WT Hu-BR2018 YFV used for the PRNTs had the envelope gene sequenced (results not included) (30), and the WT YFV envelope presented an 85% similarity compared to the 17DD strain, with a total of 228 nucleotide differences, which resulted in 27 amino acid substitutions.

### Plaque reduction neutralization test (PRNT)

For the PRNT assays, 2 × 10^5^ Vero cells were seeded in 12-well plates and cultured in high-glucose Dulbecco's Modified Eagle Medium supplemented with 5% heat-inactivated fetal bovine serum 24 h before the experiment. Serum samples were heat-inactivated (56°C for 30 minutes) before the assay. Samples were then serially diluted twofold (1:20 to 1:40,960). One hundred plaque-forming units of the vaccine 17DD or WT YFV strains were added to each serum dilution, and after a 1 h incubation at 37°C, the virus-serum suspension was used to infect Vero cells. For the infection of Vero cells, the media was removed from each well, and cells were incubated with 180 µL of virus-serum suspension for 1 h at 37°C for viral adsorption. Virus and cell controls were added to each PRNT plate. After adsorption, a semi-solid overlay, composed of 2% carboxymethylcellulose mixed with medium 199 supplemented with 2% heat-inactivated fetal bovine serum, was added to the wells and cells were incubated for 5 days at 37°C in an atmosphere with 5% of CO_2_. Plates were then fixed with 3.7% formaldehyde and stained with 1% crystal violet. The neutralizing endpoint (PRNT50) was defined as the reciprocal of the last serum dilution in which 50% of the number of plaques was reduced, compared to virus control. Assays were performed in duplicates for each serum sample. Samples with titers above 1:50 were considered positive, as specified by WHO (31).

Seropositivity rates were established for each group. Only patients with positive PRNT50 results were considered for the statistical analyses and comparisons between the groups. For all the groups, homotypic humoral immune response (YF patients against the WT strain and vaccinees against the 17DD strain) and heterotypic response (YF patients against the 17DD YFV strain and vaccinees against the WT YFV strain) were evaluated and compared. Thirteen LHep-YF patients had paired samples tested at two different time points, and the results were compared.

### Statistical analysis

Seropositivity rates, descriptive statistical analyses, geometric mean titers (GMTs), and standard deviations for nAb were established for each group. The data distribution was assessed by the Shapiro-Wilk normality test. Nonparametric Kruskal-Wallis and Mann-Whitney tests were used. The Wilcoxon comparative test was used for paired sample analysis. All statistical analyses were performed using GraphPad Prism 8.0.2 software, and values were considered statistically significant when *P* < 0.05.

## RESULTS

Demographic data analysis indicates that 86.4% of patients with YF from the study were male, aged between 18 and 79 years (mean = 49 ± 12.7 years). Male predominance was observed in all subgroups of patients, as an expected characteristic of YF outbreaks, and age varied similarly between the groups. Demographic details of the study population are described in Table 1. Detailed information regarding the participants of this study can be found in the Table S1 (https://github.com/pedrozepp/YFever).

**TABLE 1:** Demographic features and seropositivity rates of the study population, including naturally infected yellow fever patients and vaccinees

Variable/groups	Vaccinees (*n* = 23)	YF patients, total (*n* = 59)	Discharged patients (*n* = 37)	Deceased patients (*n* = 22)	NLHep-YF patients (*n* = 22)	LHep-YF patients (*n* = 15)
Gender, *n* (%)						
Male	10 (43.5)	51 (86.4)	0 (81.1)	21 (95.5)	19 (86.4)	11 (73.3)
Female	5 (21.7)	8 (13.6)	7 (18.9)	1 (4.5)	3 (13.6)	4 (26.7)
Not informed	8 (34.8)					
Age (years)						
Mean (min–max)	34 (18–59)	49 (18–79)	47(18-67)	52 (23–79)	48 (18–49)	47 (31–67)
Days after symptom onset						
Mean (min–max)	36 (31–49)	40 (4–120)	39 (30–60)	7 (4-13)	36 (30–44)	44 (31–60)
Seroprevalence, *n* (%)^*a*^						
Positive	23 (100.0)	56 (94.9)	36 (97.3)	20 (90.9)	21 (95.5)	15 (100.0)
Negative	0 (0)	3 (5.1)	1(2.7)	2 (9.1)	1 (4.5)	0 (0)

^*a*^ Calculated based on PRNT results for both 17DD and WT YFV.

The assessment of the prevalence of anti-YFV (17DD and WT) nAb among the groups of YF patients and vaccinees showed that, overall, the seropositivity rate was high among naturally infected YF patients (56 of 59, 94.9%), which was reflected in each subgroup of the study. Discharged patients presented a 97.3% seropositivity rate, with only one patient (female, 27 years old) being seronegative for YF. Two patients (male, 45 and 71 years old) were seronegative (titer <50) among the deceased group (90.9%). All patients who developed LHep-YF were seropositive after 30 days of first symptom onset (100%), and among NLHep-YF patients, only one was seronegative (95.5%). All vaccinees presented nAb anti-YF titers above the protective threshold (titer <50) 30 days after vaccination (100%). Seropositivity rates and GMT of the groups are described in Table 1.

**Fig 1:**
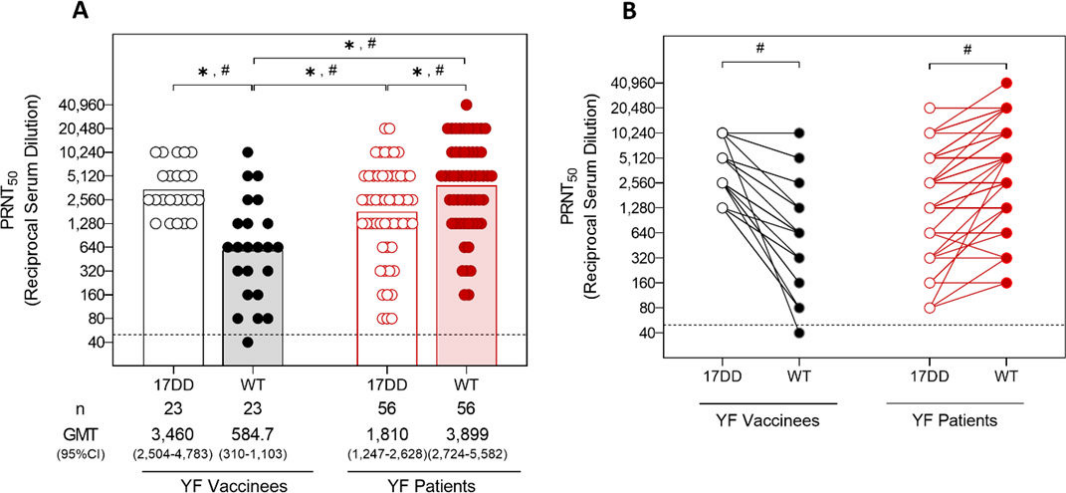
Comparison of GMTs and paired sample nAb between vaccinees and yellow fever patients. Serum samples from YF vaccinees and naturally infected YF patients were tested to measure the nAb levels against 17DD and WT YFV strains by PRNT50. Lower limit of quantification of the PRNTs were <40. Only samples with positive results were included in the analysis. The dotted line represents the protective threshold established by the WHO (1:50). GMTs were calculated for each group and compared across groups by Kruskal-Wallis and Mann-Whitney tests. Results from paired samples were compared through the Wilcoxon test. (**A**) Comparison of GMTs of vaccinees’ (in black) and YF patients’ (in red) nAb levels against the 17DD (unfilled circles) and WT (filled circles) YFV strains. Bars with asterisks (symbol for Kruskal-Wallis test) and octothorps (symbol for Mann-Whitney test) at the top of the graph indicate statistical difference, where * or # means *P* ≤ 0.05. (**B**) Comparison of nAb levels against the 17DD (unfilled circles) and WT (filled circles) YFV strains, between paired samples of vaccinees (in black) and YF patients (in red). Bars with asterisks at the top of the graph indicate statistical difference, measured by the Wilcoxon test, where # means *P* ≤ 0.05.

The analyses of the GMT obtained for the participants of this study against both WT and 17DD YFV strains showed that, overall, the groups tend to present a more prominent homotypic response compared to the heterotypic humoral response. Vaccinees presented higher levels of nAb against the 17DD strain, compared to the WT strain (Fig. 1A and B), while naturally infected patients presented higher levels of nAb against the WT strain, compared to the 17DD strain (Fig. 1A and B). Both naturally infected patients and vaccinees also responded heterotypically against the 17DD and WT YFV strains. However, vaccinees presented lower levels of nAb against the WT strain than the neutralization titers that naturally infected YF patients present against the 17DD strain (Fig. 1A). The comparison between the GMTs of vaccinees and YF naturally infected patients showed that YF patients had higher levels of nAb against the WT YFV strain, compared to vaccinees, though the humoral response of those groups against the 17DD YFV strain was similar (Fig. 1A).

**Fig 2:**
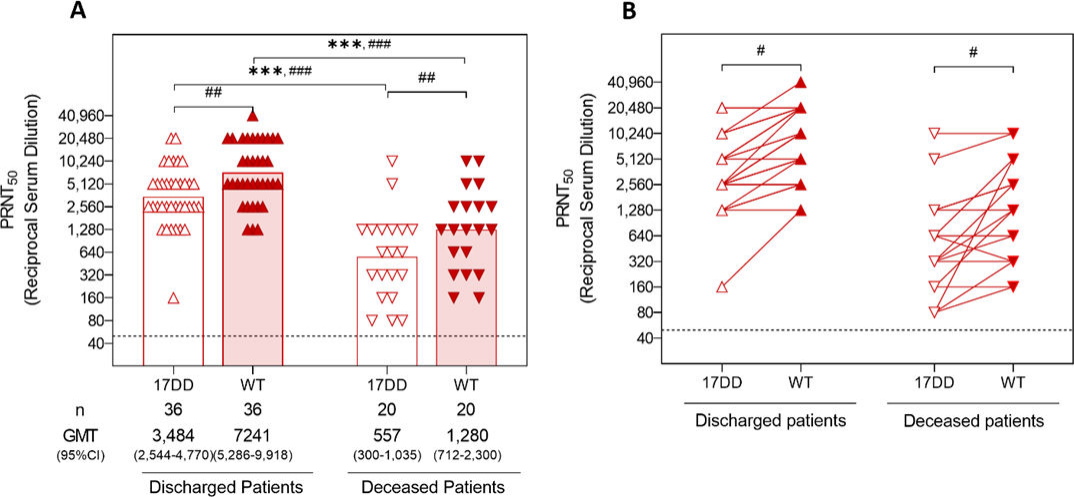
Comparison of GMTs and paired sample nAb between yellow fever patients categorized according to disease outcome of hospital discharge or death. Serum samples from naturally infected YF patients, with disease outcomes of hospital discharge (mean time between symptom onset and sample collection of 39 days) or death (mean time between symptom onset and sample collection of 7 days), were tested to measure the nAb levels against 17DD and WT YFV strains by PRNT50. LLOQ of the PRNTs was <40. Only samples with positive results were included in the analyses. The dotted line represents the protective threshold established by the WHO (1:50). GMTs were calculated for each group and compared across groups by Kruskal-Wallis and Mann-Whitney tests. Results from paired samples were compared by Wilcoxon test. (**A**) Comparison of GMTs of discharged (upward triangles) and deceased (downward triangles) patients’ (both in red) nAb levels against the 17DD (unfilled triangles) and WT (filled triangles) YFV strains. Bars with asterisks (symbol for Kruskal-Wallis test) and octothorps (symbol for Mann-Whitney test) at the top of the graph indicate statistical difference, where ## means *P* ≤ 0.01, and *** or ### means *P* ≤ 0.001. (**B**) Comparison of nAb levels against the 17DD (unfilled triangles) and WT (filled triangles) YFV strains, between paired samples of discharged YF patients (upward triangles) and deceased YF patients (downward triangles). Bar with asterisks at the top of the graph indicates statistical difference, measured by the Wilcoxon test, where # means *P* ≤ 0.05.

YF patients categorized according to disease outcome of hospital discharge or death also reflected the homotypical and heterotypical humoral response profiles displayed by naturally infected patients (higher levels of nAb against the WT strain compared to the 17DD strain) (Fig. 2A and B). Patients who were discharged from the hospital presented higher levels of nAb against the WT strain compared to the 17DD strain (Fig. 2A and B), similarly to the patients who died (Fig. 2A and B).

**Fig 3:**
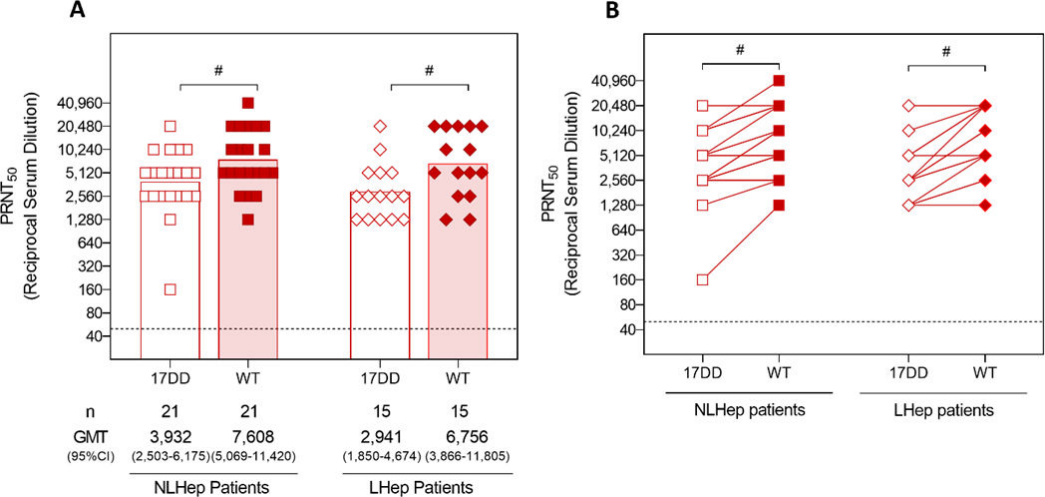
Comparison of GMTs and paired samples nAb between yellow fever discharged patients categorized according to the occurrence of late relapsing hepatitis after yellow fever. Serum samples from naturally infected YF patients, with disease outcomes LHep-YF or NLHep-YF, were tested to measure the nAb levels against 17DD and WT YFV strains by PRNT50. LLOQ of the PRNTs were <40. Only samples with positive results were included in the analyses. The dotted line represents the protective threshold established by the WHO (1:50). GMTs were calculated for each group and compared across groups by Kruskal-Wallis and Mann-Whitney tests. Results from paired samples were compared through the Wilcoxon test. (**A**) Comparison of GMTs of NLHep-YF (squares) and LHep-YF (rhombus) patients’ (both in red) nAb levels against the 17DD (unfilled squares and rhombus) and WT (filled squares and rhombus) YFV strains. Bars with octothorps (symbol for Mann-Whitney test) at the top of the graph indicate statistical difference, where # means *P* ≤ 0.05. (**B**) Comparison of nAb levels against the 17DD (unfilled squares and rhombus) and WT (filled squares and rhombus) YFV strains, between paired samples of NLHep-YF patients (squares) and LHep-YF patients (rhombus). Bar with asterisks at the top of the graph indicate statistical difference, measured by the Wilcoxon test, where # means *P* ≤ 0.05.

When YF patients who were discharged from the hospital were further categorized according to the occurrence or not of LHep-YF, the pattern of humoral response observed previously among naturally infected patients was still preserved in those subgroups. Patients without LHep-YF had higher levels of nAb against the WT strain, compared to the 17DD strain (Fig. 3A and B), as did the LHep-YF patients. No further differences were observed between these groups.

**Fig 4:**
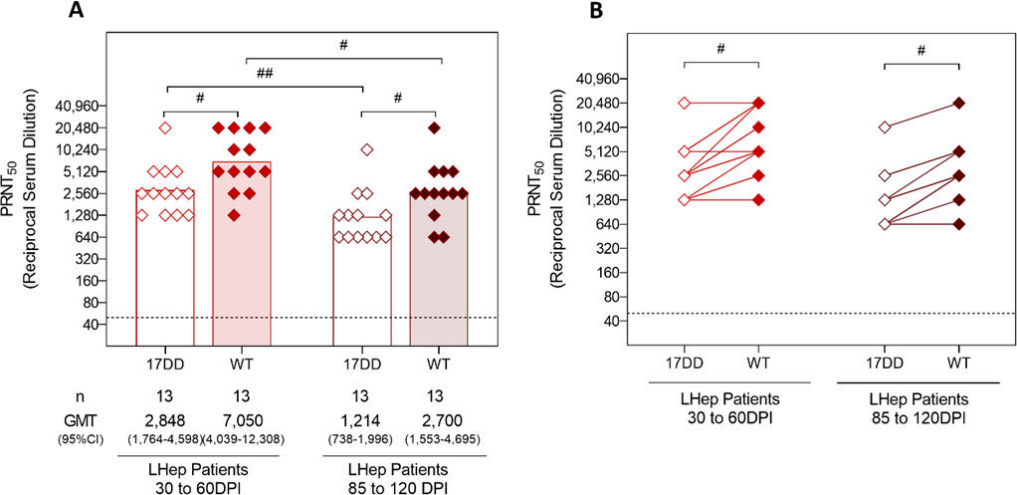
Comparison of nAb GMTs and paired comparison of nAb of LHep-YF patients between different time points. Serum samples from LHep-YF patients were collected at different time points (between 30 and 60 DPI (in red) and between 85 and 120 DPI (in dark red)) and tested to measure the nAb levels against 17DD and WT YFV strains by PRNT50. LLOQ of the PRNTs were <40. Only samples with positive results were included in the analyses. The dotted line represents the protective threshold established by the WHO (1:50). GMTs were calculated for each group and compared across time points by Kruskal-Wallis and Mann-Whitney tests. Results from paired samples were compared through the Wilcoxon test. (**A**) Comparison of GMTs of LHep-YF samples from 30/60 DPI (in red) and of LHep-YF samples from 85/120 DPI (in dark red) nAb levels against the WT (filled rhombus) and 17DD YFV (unfilled rhombus) strains. Bars with octothorps (symbol for Mann-Whitney test) at the top of the graph indicate statistical difference, where # means *P* ≤ 0.05, and ## means *P* ≤ 0.01. (**B**) Comparison of nAb levels against the 17DD (unfilled rhombus) and WT (filled rhombus) YFV strains, between paired samples of LHep-YF patients collected between 30 and 60 DPI (in red) and 85 and 120 DPI (in dark red). Bar with asterisks at the top of the graph indicates statistical difference, measured by the Wilcoxon test, where # means *P* ≤ 0.05.

Lastly, while analyzing nAb levels of 13 patients, among the YF patients who developed LHep-YF at different time points, it was possible to observe a difference in the humoral response against both WT and 17DD YFV strains, during different time points of the disease (between 30 and 60, and then 85 and 120 days after symptoms onset). Through analysis of GMT, it was possible to observe a significant decrease in response 30 days after symptom onset against both WT (Fig. 4A) and 17DD YFV strains (Fig. 4A). Through the analyses of the paired results at 30-60 and 85–120 days after symptom onset, a decrease in nAb levels was observed throughout time. However, the humoral response pattern is maintained, with the homotypic humoral response remaining higher than the heterotypic response (Fig. 4A and B).

## DISCUSSION

In the present study, homotypic and heterotypic humoral immune response profiles among patients from the 2017–2018 YF outbreak in MG, Brazil, assessed through the detection and quantification of levels of virus-specific nAb produced against the WT and 17DD strains of YFV, differed among naturally infected YF patients with different disease outcomes and vaccinees. Our data suggest that patients who recover from YF, regardless of whether they develop LHep-YF or not, generate a strong humoral immune response against the YFV, marked by extremely high levels of nAb (up to 1:40,960), especially against the homologous WT strain.

The findings of the present study demonstrated that the humoral immune response after a natural infection against the YFV is not only different but can also reach levels considerably higher than that induced by vaccination. This phenomenon was observed in the case of a WT strain from South America, whereas the 17DD vaccine is derived from an African YFV. Antigenic differences have been previously described between South American and African YF viruses (32). Consistent with this observation, individuals vaccinated with 17DD had higher nAbs against the homologous virus than against the WT strain. Our results of different immune responses against distinct strains of YFV are also corroborated by a study that evaluated virus-specific neutralization after YF vaccination in Brazilian and US individuals and demonstrated differences in the immune response by nAb against the 17DD and WT YFV in vaccinees (21).

In addition to being derived from an antigenically different (African) YFV strain, the attenuation of 17DD through a long series of empirical passages in unnatural hosts generated important differences in its genome, especially in one of the main targets of the protective immune response, the envelope protein (14, 20). The differences are even more evident when compared to viral strains from the South American genotypes due to the occurrence of genetic and biochemical changes in the envelope protein binding domains I and II, which are unique to samples circulating in South America, and they likely directly affect the recognition of those immunogenic regions by nAb (21).

Furthermore, it was also observed that the intensity of the neutralization activity against different strains of YFV varies in naturally infected individuals, according to the disease outcome. Patients who progressed to death in the early stages of the disease (first 2 weeks after symptom onset) presented a lower response mediated by nAb, when compared to samples of patients who survived, although collected at a later time point (30–60 days after symptom onset). Because no samples from the early phase of the disease were tested in the PRNT protocols for patients who survived, we cannot exclude that the differences observed here may be related to the different times of sample collection. These results should, therefore, be viewed with caution. Since the humoral immune response takes approximately 15 days to entirely develop against the YFV, as already demonstrated by studies in animal models with the 17DD strain and in previous YF outbreaks (1, 13), these findings are expected. The mechanisms that lead naturally YFV-infected individuals to evolve to death or hospital discharge have not yet been fully elucidated. It is hypothesized that failure, delay, or dysregulations in the immune response, determined by genetic, environmental, or multifactorial causes, may be related to the failure to control the infection and, consequently, to succumb to disease (24, 26, 33).

Finally, it was shown in this study that patients who developed LHep-YF, a recently described phenomenon in YF patients, presented not only high levels of nAb during the beginning of the convalescent phase but also a decrease in response against YFV after the LHep-YF onset (90–120 days after symptoms onset). There are currently only a few studies reporting the occurrence of LHep-YF (10, 12, 26, 34, 35), and to date, no studies have investigated the humoral immune response of those patients. However, a study conducted with samples from the same outbreak has shown important differences in the levels of chemokines and pro-inflammatory cytokines between LHep-YF patients and those who do not present with this complication. It was shown that patients who progress to LHep-YF presented a dichotomic profile of serum soluble mediators at acute and convalescent stages, displaying lower levels of chemokines (CCL11, CXCL8, and CXCL10) and pro-inflammatory cytokines (IL-1β, TNF-α, IFN-γ, and IL-17) at acute stage of the disease, compared to patients who did not develop this condition, and increased levels of most soluble mediators at convalescent disease. Patients who did not progress with LHep-YF also displayed a more complex serum mediator network than those who developed LHep-YF. This inability of LHep-YF patients to build a robust response during the acute infection was suggested to be associated with the persistence of the YFV (26). Because this phenomenon was only recently reported, first described during the 2017–2018 YF outbreak in southeastern Brazil, further studies are still needed to better understand viral dynamics and precise immune mechanisms associated with its occurrence.

Even though we can find a significant number of published papers demonstrating the role of most components of the innate and adaptive immune responses against YFV, most studies have been done using the vaccine YFV strains (17D and 17DD), an attenuated viral sample similar to the WT strain, as a study model (20). Currently, little is known about the range of the immune response developed during natural infections of YFV. There is still a significant knowledge gap concerning the immune response in people living in endemic regions for YF (18). Although a safe and effective vaccine against YF exists and despite the current advances in studies related to YF, there is still a need to expand and update our understanding of the pathogenic and immunological mechanisms of this disease, especially in natural infections since the clinical spectrum of YF is directly related to immunological features of the host, in addition to viral characteristics (18).

It is important to point out that the present study may present some limitations, such as a small sample size, limited records of vaccine documentation for the YF patients, and the absence of follow-up points for most participants. The study population also presented an uneven male/female distribution, with a male predominance observed among all groups evaluated. Although this difference in gender distribution is not ideal in most serological studies, the male predominance is consistent with the routinely observed higher incidence of YF in men, which is associated with already known risk factors such as proximity to forest areas for labor or recreation, and the fact that they are more likely to refuse vaccination (36). Furthermore, studies have shown that, although some aspects of the immune response against the YFV can be higher in females compared to males, differences in the humoral immune response related specifically to sex have not yet been reported for yellow fever patients or vaccinees (37).

In addition, the PRNT was the only method employed in this study to assess the humoral immune response of the patients from the 2016–18 outbreak and in vaccinees. While alternative methodologies exist, such as the enzyme-linked immunosorbent assay for IgM (MAC-ELISA) and hemagglutination inhibition assays, and are commonly used, they often require corroborative testing due to their suggestive nature. Despite being labor intensive and time consuming, PRNT assays stand out for their superior sensitivity, specificity, and accuracy, making them the preferred choice for serological diagnosis and the best correlate of protection against yellow fever, according to the WHO (25, 38). Furthermore, we opted against using other methods due to concerns regarding their heightened cross-reactivity and limited resources, given that this study was conducted during an outbreak. Finally, we did not have access to the WHO YF standard serum, which would have allowed the standardization of the results and direct comparison of the data obtained here with other serological surveys. Some of those issues will already be addressed by a systemic immune response investigation, already set up by our study group, for patients from the same YF outbreak.

In conclusion, the unique yet small data set of samples used in this study provided additional evidence that the immune responses triggered by the vaccine and WT strains of the YFV are different and contributed to a better understanding of the immune humoral response in naturally infected YF patients, with different disease outcomes. Furthermore, these findings are important for control and immunization programs in endemic areas since previous infection by the YFV might significantly impact disease management and epidemiological studies.
